# The inhibition of fat cell proliferation by *n-3 *fatty acids in dietary obese mice

**DOI:** 10.1186/1476-511X-10-128

**Published:** 2011-08-02

**Authors:** Michal Hensler, Kristina Bardova, Zuzana Macek Jilkova, Walter Wahli, Daniel Meztger, Pierre Chambon, Jan Kopecky, Pavel Flachs

**Affiliations:** 1 Department of Adipose Tissue Biology, Institute of Physiology Academy of Sciences of the Czech Republic v.v.i., Prague, Czech Republic; 2 Center for Integrative Genomics, National Research Center Frontiers in Genetics, University of Lausanne, Lausanne, Switzerland; 3 Department of Functional Genomics, Institute of Genetics and Molecular and Cellular Biology, Illkirch, France

**Keywords:** DHA and EPA, fish oil, fat cell turnover

## Abstract

**Background:**

Long-chain n-3 polyunsaturated fatty acids (LC n-3 PUFA) of marine origin exert multiple beneficial effects on health. Our previous study in mice showed that reduction of adiposity by LC *n-3 *PUFA was associated with both, a shift in adipose tissue metabolism and a decrease in tissue cellularity. The aim of this study was to further characterize the effects of LC *n-3 *PUFA on fat cell proliferation and differentiation in obese mice.

**Methods:**

A model of inducible and reversible lipoatrophy (aP2-Cre-ER^T2 ^PPARγ^L2/L2 ^mice) was used, in which the death of mature adipocytes could be achieved by a selective ablation of peroxisome proliferator-activated receptor γ in response to i.p. injection of tamoxifen. Before the injection, obesity was induced in male mice by 8-week-feeding a corn oil-based high-fat diet (cHF) and, subsequently, mice were randomly assigned (day 0) to one of the following groups: (i) mice injected by corn-oil-vehicle only, i.e."control" mice, and fed cHF; (ii) mice injected by tamoxifen in corn oil, i.e. "mutant" mice, fed cHF; (iii) control mice fed cHF diet with15% of dietary lipids replaced by LC *n-3 *PUFA concentrate (cHF+F); and (iv) mutant mice fed cHF+F. Blood and tissue samples were collected at days 14 and 42.

**Results:**

Mutant mice achieved a maximum weight loss within 10 days post-injection, followed by a compensatory body weight gain, which was significantly faster in the cHF as compared with the cHF+F mutant mice. Also in control mice, body weight gain was depressed in response to dietary LC *n-3 *PUFA. At day 42, body weights in all groups stabilized, with no significant differences in adipocyte size between the groups, although body weight and adiposity was lower in the cHF+F as compared with the cHF mice, with a stronger effect in the mutant than in control mice. Gene expression analysis documented depression of adipocyte maturation during the reconstitution of adipose tissue in the cHF+F mutant mice.

**Conclusion:**

Dietary LC *n-3 *PUFA could reduce both hypertrophy and hyperplasia of fat cells *in vivo*. Results are in agreement with the involvement of fat cell turnover in control of adiposity.

## Background

Adipose tissue and its secreted products, adipokines, have a major role in the development of obesity-associated metabolic disarrangement including dyslipidaemia and insulin resistance (i.e. the components of metabolic syndrome). Long-chain *n-3 *polyunsaturated fatty acids (LC *n-3 *PUFA), namely eicosapentaenoic acid (EPA; 20:5 n-3) and docosahexaenoic acid (DHA; 22:6 n-3) act as natural hypolipidemics, reduce risk of cardiovascular disease and could prevent development of obesity and insulin resistance in humans [1]. Also our experiments on mice have demonstrated that substitution of 15% lipids in a corn oil-based high fat diet (cHF) by LC *n-3 *PUFA concentrate (i.e. feeding cHF+F diet, see Methods) prevented dietary induced obesity and associated metabolic disorders [2-4]. The preferential decrease in abdominal adipose tissue growth resulted not only from modulation of metabolism in response to LC *n-3 *PUFA [5], but probably also in part from the inhibition of fat cell proliferation. Quantification of adipose tissue DNA revealed that the reduction of epididymal fat was associated with 34-50% depression of tissue cellularity [2]. *In vitro*, both EPA and DHA inhibited adipocyte differentiation and lipid droplet formation [6-8] and DHA induced apoptosis in postconfluent preadipocytes [7]. To further characterize inhibitory effect of LC *n-3 *PUFA on adipose cell proliferation and differentiation *in vivo*, and to learn more about the role of fat cell turnover in the control of adiposity [9], we used mouse transgenic model of inducible and reversible lipoatrophy (aP2-Cre-ER^T2 ^PPARγ^L2/L2^). In this model, death of mature brown and white adipocytes is achieved by selective ablation of peroxisome proliferator-activated receptor γ (PPARγ) using the tamoxifen-dependent Cre-ER^T2 ^recombination system [10]. PPARγ is essential for survival of mature adipocyte and deletion of PPARγ causes adipocyte death, triggers an inflammatory reaction and promotes proliferation and differentiation of preadipocytes into new adipocyte expressing PPARγ [10]. Our results document that LC *n-3 *PUFA slow down compensatory adipose tissue growth and adipocyte proliferation after the transgenically-induced transient lipoatrophy and they also support the notion [9] that fat cell turnover is involved in the control of adipose tissue mass.

## Methods

### Animals and experimental design

**Figure 1:**
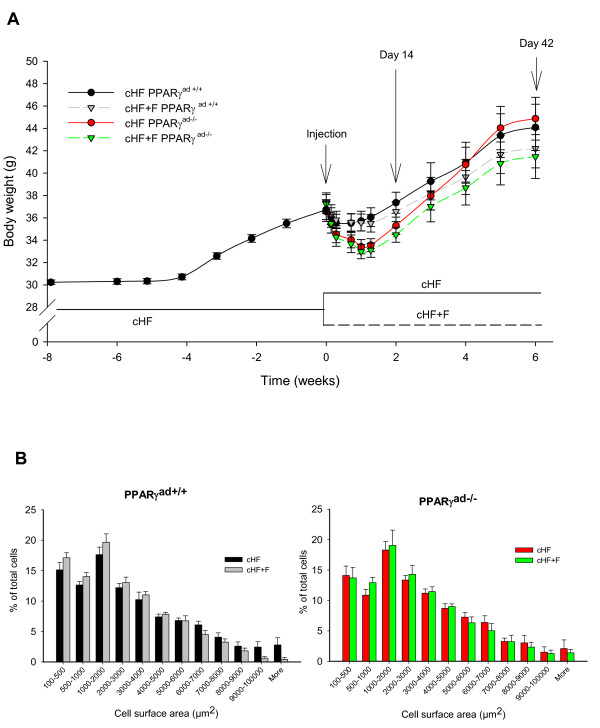
**Growth characteristics**. After 8 weeks of high-fat (cHF) feeding, mice were randomly assigned to one of the following groups: (i) control mice, fed cHF (cHF PPARγ^ad+/+^); (ii) mutant mice, fed cHF (cHF PPARγ^ad-/-^); (iii) control mice, fed cHF enriched by LC *n-3 *PUFA (cHF+F PPARγ^ad+/+^); and (iv) mutant mice, fed cHF+F (cHF+F PPARγ^ad-/-^). Part of mice were killed at day 14, while the remaining mice were killed at day 42. **A **Body weights; **B **Frequency distribution of adipocyte cell surface area in epididymal fat at day 42. Data are means ± SE; n = 10.

Adipose tissue specific PPARγ conditional knock out mice (aP2-Cre-ER^T2 ^PPARγ^L2/L2^) were used [10]. In this transgenic model, PPARγ can be selectively ablated in mature adipocytes by the conditional Cre-ER^T2 ^Cre recombinase whose activity depends on tamoxifen administration [10]. At 4 weeks of age, male mice were weaned onto a standard laboratory chow (Chow; lipid content ~3.4% wt/wt; extruded R/M-H diet; Ssniff Spezialdiäten, Soest, Germany) and maintained at 22°C on a 12 h light-dark cycle with free access to food and water. Starting at 3 months of age, mice were fed a corn oil based high-fat diet (cHF; lipid content ~35% wt/wt), and at 5 months of age (day 0; see Figure 1) the animals were randomly assigned to one of the following groups: **(i) **premutant aP2-Cre-ER^T2 ^PPARγ^L2/L2 ^mice, intraperitoneally twice injected by 100 μl of corn oil at day 0 and day 1, hereafter named **control PPARγ ^ad+/+ ^**mice, which were fed cHF diet; **(ii) **aP2-Cre-ER^T2 ^PPARγ^L-/L- ^mice intraperitoneally twice injected by 1 mg tamoxifen in 100 μl of corn oil at day 0 and day 1, and hereafter named **mutant PPARγ ^ad^**^-/- ^mice, which were fed cHF diet; **(iii) **control PPARγ^ad+/+ ^mice fed cHF diet supplemented with LC *n-3 *PUFA concentrate (product EPAX 1050 TG (46% DHA, 14% EPA), EPAX, a.s., Aalesund, Norway) replacing 15% of dietary lipids (cHF+F diet); and **(iv) **mutant PPARγ ^ad-/- ^mice fed cHF+F diet. Samples were collected for various analyses at day 14 and at day 42. Fresh rations of food were distributed every second day, food consumption and body weights were recorded. Mice were sacrificed by cervical dislocation in random fed state (between 8 a.m. and 10 a.m.), and EDTA-plasma and selected tissues were collected for various analyses. Several independent experiments were performed, in accordance with the guidelines of the Institute of Physiology for the use and care of laboratory animals.

### Histological analysis of adipose tissue

Epididymal white fat samples were fixed in 4% formaldehyde embedded in paraffin and stained with hematoxylin/eosin. Morphometry of adipocytes was performed as before [11], using NIS-Elements 3.0 AR morphometric software (Laboratory Imaging, Prague, Czech Republic). The morphometry data are based on more than 800 cells taken randomly from six different areas per animal.

### Quantitative real time PCR

Total RNA isolated using TRI Reagent (Molecular Research Center, Inc, Cincinnati, OH, USA). Levels of various transcripts were evaluated using LightCycler 480 II instrument (Roche Diagnostic Ltd., Rotkreuz, Switzerland) and LightCycler 480 SYBR Green I Master kit (Roche Diagnostic Ltd., Mannheim, Germany). PCR condition were 95°C for 5 min and 45 cycles of 95°C for 10 s, 55-60°C for 10 s and 72°C for 20 s. Specificity of the amplified PCR product was assessed by performing a melting curve analysis. Lasergene 7 software (DNASTAR, Inc. Madison, WI, USA) was used to design primers. The PCR primer pairs were used as in Table 1. To correct for intersample variation, levels of the transcript were normalized using geometrical mean of two reference genes.

**Table 1:** Gene specific forward and reverse primer sequences used for qRT-PCR

Gene	Forward primer	Reverse primer	NCBI accession number
*Cidec/Fsp27*	GACAAGCCCTTCTCCCTGGTG	CCATCAGAACAGCGCAAGAAGAGA	NM_178373.3
*Cox3*	TCATCGTCTCGGAAGTATTTTT	CCACATAAATCAAGCCCTACTAAT	NC_005089.1
Cyph β*	ACTACGGGCCTGGCTGGGTGAG	TCATCATTGTCGACTCCGGCA	NM_011149.2
Eef2*	GAAACGCGCAGATGTCCAAAAGTC	CCTTAGACTTGCAGCCCGGC	NM_007907.2
*Pgc-1α*	CCCAAAGGATGCGCTCTCGTT	AATCAAGCCACTACAGACACCGCA	NM_008904.2
*Pparα*	TGCGCAGCTCGTACAGGTCATCAA	TAAGACTACCTGCTACCGAAATGGGGG	NM 011144.6
*Scd-1*	ACTGGGGCTGCTAATCTCTGGGTGTA	TAACAAACCCACCCCAGAGATAAAGCC	NM_009127.4

*Cidec/Fsp27 - *cell death-inducing DFFA-like effector c; *Cox3 - *cytochrome c oxidase subunit III; *Cyph β - *cyclophilin-β; *Eef2 - *eukaryotic translation elongation factor 2; *Pgc-1α - *PPARγ coactivator 1α; *Pparα - *peroxisome proliferator-activated receptor α; *Scd-1 *- stearoyl- Coenzyme A desaturase 1; *- reference gene.

### Plasma adiponectin

Adiponectin levels were determined using Western blotting as before [11]. Tris-acetate gradient gel (NuPAGE 3-8%, Invitrogen, Life Technologies, Carlsbad, CA, USA) was used for division of multimeric forms of adiponectin in plasma. Primary rabbit anti-mouse polyclonal antibodies (BioVendor, Brno, Czech Republic), followed by secondary donkey anti-rabbit IgG infrared dye conjugated antibodies (IR Dye 800, Rockland, Gilbertsville, PA, USA) were using. Membranes were scanned using Odyssey IR imager (Li-Cor Biosciences, Lincoln, NE, USA).

### Statistical analysis

All values are presented as mean ± SE. Logarithmic transformation was used to stabilize variance in cells when necessary. Data were analyzed by one-way ANOVA with Holm-Sidak posthoc test using SigmaStat 3.5 statistical software. Comparisons were judged to be significant at p ≤ 0.05.

## Results

After 8 weeks of cHF feeding, obese mice (body weight 36.9 ± 0.4 g) were randomly assigned to one of the four experimental groups (day 0; see Methods and Figure 1). In accordance with the previous study on this transgenic model [10], temporary controlled PPARγ ablation caused a transient body weight loss reflecting changes in body fat content. Mutant PPARγ^ad-/- ^mice (i.e., mice injected by tamoxifen in corn oil) achieved a maximum weight loss within 10 days post-injection, independent of diet. Also control PPARγ ^ad+/+ ^mice showed a small reduction of body weight, reflecting the stress associated with the corn oil (without tamoxifen) injection. Afterward, all mice started to gain body weight with a different dynamics in various groups. Thus, (i) the cHF mice gained body weight much faster than the cHF+F mice, (ii) at 6 weeks after the injection, when body weights stabilized in a group-specific manner, body weights of the cHF mice were bigger as compared with the cHF+F mice; and (iii) the dissociation in final body weights in response to the presence/absence of LC *n-3 *PUFA in the diet was more apparent in the mutant as compared with the control mice. Therefore, the mutant mice showed the highest final body weights when fed cHF diet, and the highest reduction in body weight gain when fed cHF+F diet (Figure 1A and Table 2). In any period post-injection, food consumption was not affected by either dietary LC *n-3 *PUFA or genotype (not shown). The weights of interscapular brown fat, dorsolumbar subcutaneous and epididymal white fat were significantly reduced in mutant mice killed at day 14 (Table 2). At day 42, there were no differences between control and mutant mice within same diets, while cHF+F diet became the main force of reduction of adiposity. In accordance with its effect on body weight gain, the cHF+F diet prevented white fat depot growths between day 14 and day 42 more effectively in the mutant as compared with control mice (Table 2).

**Table 2:** Body weight, fat depots weight, adipocyte size and plasma adiponectin

	PPARγ^ad+/+^		PPARγ^ad-/-^	
	cHF	cHF+F	cHF	cHF+F
**Final body weight **(g)				
Day 42	44.1 ± 2.1	42.2 ± 1.8	44.9 ± 1.9	41.5 ± 2^c^
				
**Epididymal fat **(mg)				
Day 14	1607 ± 210	1369 ± 165	1169 ± 184^a^	1180 ± 28^a^
Day 42	2934 ± 292	2391 ± 191^a^	2892 ± 354	2378 ± 261^a,c^
				
Size of adipocytes (μm^2^)				
Day 14	4397 ± 518	3381 ± 285^a^	3471 ± 422^a^	3126 ± 323^a,b^
Day 42	3232 ± 267	2653 ± 139	3182 ± 323	2964 ± 273
				
**Subcutaneous fat **(mg)				
Day 14	479 ± 59	395 ± 39	319 ± 37^a^	332 ± 37^a^
Day 42	686 ± 64	542 ± 59	656 ± 73	463 ± 74^a,c^
				
**Interscapular brown fat **(mg)				
Day 14	147 ± 17	124 ± 11	123 ± 13^a^	121 ± 8^a^
Day 42	190 ± 23	159 ± 13	179 ± 15	150 ± 3
				
**Adiponectin **(A.U.)				
Day 14				
HMW	0.70 ± 0.06	0.79 ± 0.06	0.52 ± 0.03^a^	0.50 ± 0.04^a,b^
MMW	0.79 ± 0.04	0.82 ± 0.03	0.51 ± 0.04^a^	0.57 ± 0.02^b^
LMW	0.01 ± 0.00	0.01 ± 0.00	0.01 ± 0.00	0.01 ± 0.00
Total	1.49 ± 0.10	1.62 ± 0.08	1.04 ± 0.06^a.b^	1.12 ± 0.06^a,b^
				
Day 42				
HMW	0.54 ± 0.04	0.96 ± 0.10^a^	0.66 ± 0.07	1.06 ± 0.11^a,c^
MMW	0.53 ± 0.02	0.66 ± 0.05	0.58 ± 0.04	0.75 ± 0.05^a,c^
LMW	0.01 ± 0..0	0.02 ± 0.0	0.02 ± 0.0	0.02 ± 0.0
Total	1.09 ± 0.06	1.64 ± 0.15^a^	1.25 ± 0.10	1.82 ± 0.14^a,c^

A.U., arbitrary units; HMW, high molecular weight adiponectin; MMW, medium molecular weight adiponectin; LMW, low molecular weight adiponectin; data are means ± SE; n = 10; *a, b, c - *significant differences compared to cHF PPARγ^ad+/+^, cHF+F PPARγ^ad+/+ ^and cHF PPARγ^ad-/-^, respectively.

Histological and morphometric analysis of epididymal fat was performed to characterize effect of PPARγ ablation on tissue morphology in the model of dietary obese mice. At day 14, mean size of adipocytes was smaller in mutant as compared with control mice, and it was decreased further in response to dietary LC *n-3 *PUFA, resulting in the smallest adipocytes in the cHF+F mutant mice (Table 2). Importantly, at day 42, mean size of adipocytes was similar in all the groups (Table 2), and also distribution of fat cell sizes was not significantly affected by either diet, or the transient genetic ablation of PPARγ (Figure 1B).

**Figure 2:**
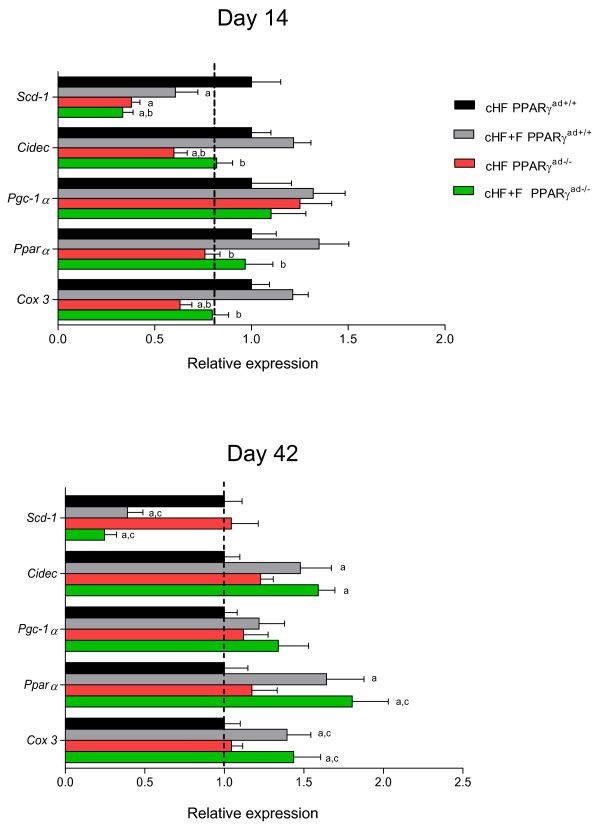
**Analysis of gene expression in epididymal fat**. Transcript levels were measured using qRT-PCR in total RNA isolated from epididymal fat at day 14 or day 42. *Scd-1*, stearoyl-Coenzyme A desaturase 1; *Cidec*, cell death-inducing DFFA-like effector c; *Pgc-1α*, peroxisome proliferative activated receptor γ, coactivator 1 alpha; *Pparα*, peroxisome proliferator activated receptor α; *Cox3*, cytochrome c oxidase subunit III. Data are means ± SE; n = 10; *a, b, c - *significant differences compared to cHF PPARγ^ad+/+^, cHF+F PPARγ^ad+/+^, and cHF PPARγ^ad-/-^, respectively.

Expression of selected genes was quantified in epididymal fat (Figure 2). At day 14, compared to cHF control mice, a marked down-regulation of *Scd-1 *was found in remaining groups. At day 42, only dietary LC *n-3 *PUFA diminished *Scd-1 *expression independently on genotype. Genes connected to lipid metabolism, *Pparα, Cox3 *and *Cidec (Fsp27)*, were transiently down-regulated in the mutant as compared with control mice at day 14 only. Dietary LC *n-3 *PUFA induced expression of these genes at both time points, with the strongest effects elicited in the mutant mice in response to the longer treatment.

Plasma levels of total adiponectin and of its biological active high molecular weight (HMW) form, which is implicated in enhancement of insulin sensitivity [12], were lower in mutant as compared with control mice at day 14. Latter on, total and HMW adiponectin level were similar in both control and mutant mice, with a tendency to be higher in the mutant mice (Table 2). In accordance with our previous findings showing induction of adiponectin by LC *n-3 *PUFA [3,13], adiponectin levels were higher in the cHF+F as compared with the cHF mice, in both control and mutant mice, even at day 14 (Table 2).

## Discussion

Adipose tissue belongs to the most flexible tissues in the body, because it is rapidly remodeled by hyperplasia and hypertrophy of adipocytes, depending on energy fluxes. Fat cell turnover might contribute to determination of adiposity [9]. However, the mechanisms, which are involved in setting the turnover and their links to energy balance, remain to be established. Enormous plasticity of adipose tissue was also documented by our results. In agreement with the first study on this transgenic model [10], as well as other model of reversible lipoatrophy [14], 6 weeks after tamoxifen injection, adipose tissue from mutant mice appeared histologically identical to untreated mice, indicating complete tissue regeneration. The global, robust return of fat mass was confirmed by progressive body weight gain, especially in mutant mice fed cHF. The death fat cells are replaced by newly differentiated adipocytes originated from endogenous preadipocytes. The histological changes of epididymal fat depot from mice fed cHF were mirrored by changes of transcript levels for genes encoding *Scd-1, Cidec (FSP27)*, and *Cox3*. As already described, the above genes are activated during adipocyte differentiation and maturation [15-17]. Similarly, plasma level of adiponectin was associated with the reduction of fat mass in the mutant mice compared to controls at day 14. Thus, similarly as in the transgenic mice fed chow diet [10], also in the dietary obese mice studied here, the transgenically-induced lipoatrophy was fully reversible within approximately 6 weeks after the PPARγ ablation.

Based on our previous results suggesting that the anti-obesity effect LC *n-3 *PUFA could be, at least in part, explained by the prevention of fat cell proliferation during high-fat diet-feeding, we have challenged both control and the transgenic mice by dietary LC *n-3 *PUFA. Indeed, the cHF+F diet treatment decreased body weight gain in both control and mutant mice. At both time points analyzed (day 14 and day 42), total body weight, as well as weights of adipose depots were the lowest in the mutant mice fed cHF+F diet. Nevertheless, when body weights stabilized in a group-specific manner, by the end of the treatment at 6 weeks, body weights were affected more by the diet than by the transient genetic ablation. Equal size of adipocytes in all the groups at the end of the treatment, suggest (i) similar number of adipocytes in epididymal fat within the same type of diet; (ii) existence of mechanisms, which tend to stabilize fat cell number via controlling fat cell turnover; and (iii) tuning of these hypothetical mechanisms by LC *n-3 *PUFA or their metabolites (see below).

In accordance with our already published data showing stimulation of mitochondrial biogenesis and β-oxidation [5] in white adipose tissue in response to dietary LC *n-3 *PUFA, feeding cHF+F diet induced *Pparα *and *Cox3 *expression in control mice. At day 14, tamoxifen-induced-lipoatrophy in mutant mice was associated with lower expression of the above genes and interfered also with the LC *n-3 *PUFA effects. At day 42, expression of *Pparα *and *Cox3 *was strongly induced by dietary LC *n-3 *PUFA while no effect of tamoxifen injection was found. Furthermore, qRT-PCR analysis shown identical pattern of *Cidec *expression, i.e. significant reduction by tamoxifen treatment in control mice at day 14 and marked induction by LC *n-3 *PUFA at day 42 with the highest level in the mutant cHF+F mice.

Our results document that dietary LC *n-3 *PUFA could decrease adiposity in obese mice by a mechanism, which depends on counteraction of both, differentiation and proliferation of adipose cells. One possible mechanism involves the changes in fatty acid composition of cellular membranes, and hence, altered formation of PUFA-derived active metabolites like as eicosanoids [1,6,18,19]. The anti-proliferative effect may be involved in the decreased adiposity of pups born to rat dams that fed diets supplemented by *n-3 *fatty acids during gestation and sucking (reviewed in ref. [20]). It has been hypothesized that LC *n-3 *PUFA are involved in the anti-obesity effect of breast-feeding [21]. Importantly, our results support the notion [9] that adiposity is closely linked to the control of fat cell turnover and that mechanisms could exist, which control fat cell proliferation independent of energy balance.

## Abbreviations

cHF: corn oil-based high fat diet; cHF+F: cHF diet supplemented with LC *n-3 *PUFA concentrate (15% of dietary lipids); DHA: docosahexaenoic acid; EPA: eicosapentaenoic acid; PPAR-α (-γ): peroxisome proliferator activated receptor-α (-γ); LC *n-3 *PUFA: long-chain *n-3 *polyunsaturated fatty acids.

## Competing interests

The author declares that they have no competing interests.

## Authors' contributions

MH performed the most of experiments and statistical analysis, contributed to writing of the manuscript; ZMJ and KB performed the histological analysis; WW, DM and PC created aP2-Cre-ER^T2 ^PPARγ^L2/L2 ^transgenic murine model, participated in coordination of the study and revising the manuscript; JK participated in the design and coordination of the study and contributed to writing of the manuscript; DM and PC participated in the design and coordination of the study; PF performed initial animal experiments, participated in design and coordination of the study and drafted the manuscript. All authors read and approved the final version of the manuscript.
